# Highly flexible method for the fabrication of photonic crystal slabs based on the selective formation of porous silicon

**DOI:** 10.1186/1556-276X-7-449

**Published:** 2012-08-09

**Authors:** Gonzalo Recio-Sánchez, Zhiya Dang, Vicente Torres-Costa, Mark BH Breese, Raul-Jose Martín-Palma

**Affiliations:** 1 Departamento de Física Aplicada, Universidad Autónoma de Madrid, Avda. Francisco Tomás y Valiente 7, Cantoblanco, Madrid, 28049, Spain; 2 Center for Ion Beam Applications (CIBA), Department of Physics, National University of Singapore, Singapore, 117542, Singapore; 3 Singapore Synchrotron Light Source (SSLS), 5 Research Link, National University of Singapore, 5 Research Link, Singapore, 117603, Singapore

**Keywords:** Photonic slabs, NanoPSi, Photonic band structure, Proton beam writing

## Abstract

A novel fabrication method of Si photonic slabs based on the selective formation of porous silicon is reported. Free-standing square lattices of cylindrical air holes embedded in a Si matrix can be achieved by proton beam irradiation followed by electrochemical etching of Si wafers. The photonic band structures of these slabs show several gaps for the two symmetry directions for reflection through the z-plane. The flexibility of the fabrication method for tuning the frequency range of the gaps over the near- and mid-infrared ranges is demonstrated. This tunability can be achieved by simply adjusting the main parameters in the fabrication process such as the proton beam line spacing, proton fluence, or anodization current density. Thus, the reported method opens a promising route towards the fabrication of Si-based photonic slabs, with high flexibility and compatible with the current microelectronics industry.

## Background

Since the concept of photonic crystal was proposed and theoretically discussed several decades ago [1,2], many novel photonic devices have been proposed aiming at controlling the propagation of electromagnetic waves. Practical applications of these results require the use of three-dimensional (3D) photonic crystal devices with 3D band gaps [3,4]. However, the fabrication of such structures is not a simple task since they require a complex 3D connectivity and strict alignment requirements [5]. An alternative aiming at an easier fabrication process relies on the development of photonic crystal slabs. These are periodic two-dimensional dielectric structures which use index guiding to confine light in the third dimension [6-8]. Photonic crystal slabs share almost of the properties with true 3D photonic crystals; however, new issues such as slab thickness or mirror symmetries are determinant in their optical behavior [9].

Additionally, Si-based photonic crystals are one of the most promising photonic devices due to their easy integration in Si technology, allowing novel applications in several fields, such as optical devices including waveguides and filters [10,11], or in the field of telecommunications, as antenna substrates or reflectors [12]. A wide variety of methods have been used to fabricate photonic crystal slabs based on Si. Semiconductor fabrication techniques such as advanced lithography or holography [13,14] are some of the most widely used methods. Also, nanoindentation lithography followed by macroporous silicon formation is one of the most recently used techniques to fabricate Si-based photonic crystals in two and three dimensions [15].

Nanostructure porous silicon (nanoPSi) is a popular material for the fabrication of photonic devices since it shows a variety of interesting properties such as efficient photoluminescence in the visible range at room temperature [16], tunable refractive index, or low light absorption in the visible [17]. Hence, nanoPSi has been used for the fabrication of a number of optical devices including one- [18,19] and two-dimensional [20,21] photonic crystals. These devices allow novel applications such as bimolecular screening [22], amplification of optical detection [23], and encoded microcarriers [24].

In the present work, a novel and highly flexible fabrication process of Si-based photonic crystal slabs is demonstrated. This method is based on the selective formation of nanoPSi by electrochemical etching after a proton beam with different energies and fluences is focused on the Si wafer. This technique allows fabrication of free-standing slabs consisting of square lattices of cylindrical air holes in a Si matrix which can work over most of the near- and mid-infrared range. The flexibility of this technique for tuning the frequency ranges and sizes of the photonic gaps is demonstrated and makes this method a very promising candidate for the development of Si-based photonic devices. On one hand, this flexibility is given by the possibility of adjusting the lattice parameter of the structures, by changing the spacing between irradiated lines. This is easily achievable due to the special experimental setup, which allows focusing the proton beam down to 100 nm, permitting a course tuning of the frequency range of the gaps. On the other hand, by adjusting the main parameters in the fabrication process such as proton fluence or anodization current density, the radius of the air holes and slab thickness can be modified in a very accurate way, given the possibility of a fine tuning of the gaps.

## Methods

**Figure 1:**
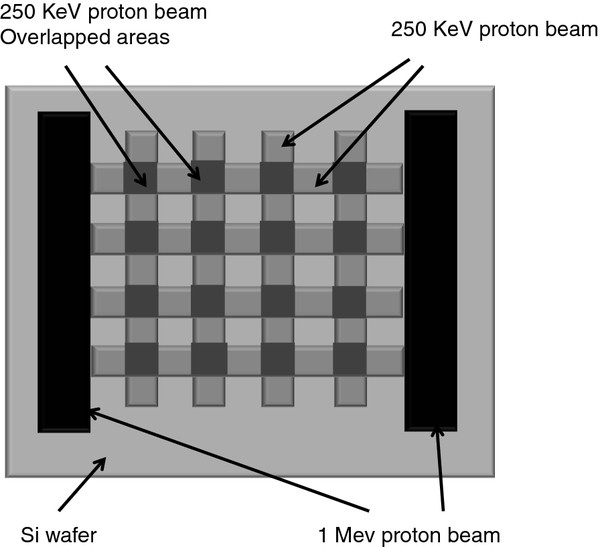
Schematics of vacancy distribution at 2.4-μm depth after proton beam irradiations on Si surface.

Photonic slabs consisting of a square lattice of cylindrical air holes in a Si matrix were fabricated in several steps as follows: First, a 250-keV proton beam was focused down to 100 nm and scanned in both directions to define a square grid on the surface of a p^+^-type Si wafer (orientation <100>; resistivity 0.02 Ω cm). For 250-keV protons, high-defect regions are generated at a depth of 2.4 μm in bulk Si, after lines were irradiated horizontally and vertically with moderate line fluence, as Figure 1 shows. The proton beam was provided by a nuclear microprobe at the Center for Ion Beam Applications, National University of Singapore. Setup of this equipment allows the control of beam line spacing and proton fluence.

In order to obtain a free-standing structure, a high-energy proton beam of 1 MeV, which has a deeper penetration in the Si wafer with an extremely high fluence of 1 × 10^12^/cm, was used to define supports at the same area, as Figure 1 presents.

**Figure 2:**
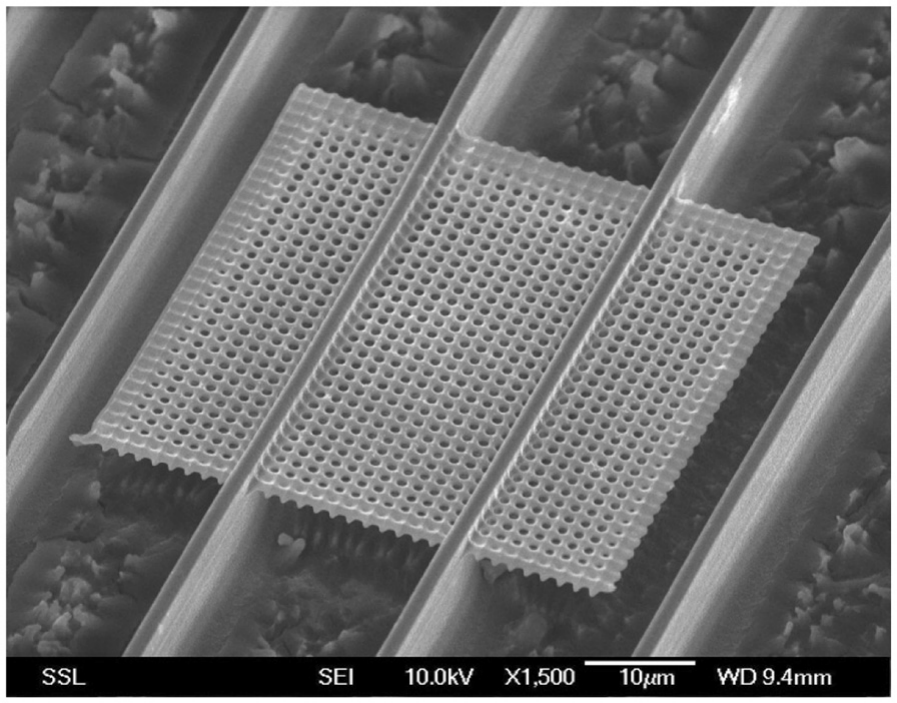
** Scanning electron microscope image of the resulting free-standing Si-based photonic slab.** The slab is a square mesh of cylindrical air holes in a Si matrix.

After the irradiation of the Si wafers, nanoPSi is selectively formed by the electrochemical etch in HF (48%):EtOH (98%) (1:1) solutions. NanoPSi grows in low-defect regions and unirradiated zones, whereas high-defect regions remain as Si. Due to the higher defect density attributable to overlapped areas at the intersection of irradiated lines, circular holes instead of square holes of nanoPSi are formed. After removal of nanoPSi in KOH solutions, a free-standing slab of 2D square lattice of air holes is obtained, as Figure 2 shows.

An important development in Si photonic is the ability of using deep localized defects at the end of the range of high-energy protons. This allows machining 3D Si structures within bulk Si by selective formation of nanoPSi in the subsequent anodization process [25,26]. As the proton beam penetrates the semiconductor, it damages the Si crystal by producing additional vacancies [27]. Vacancy distribution produced in bulk Si depends on the energy and fluence of the proton beam. Then, the irradiated wafer is electrochemically anodized in a dilute HF solution as mentioned above. At moderate fluence, the buried regions of high vacancy concentration inhibit nanoPSi formation, whereas regions with low-density vacancies or unirradiated zones allow nanoPSi formation. Finally, nanoPSi can be removed in KOH solutions.

A Hitachi S-3000 N scanning electron microscope (SEM; Hitachi, Ltd., Chiyoda, Tokyo, Japan) with conventional thermionic filament was used to characterize the structures.

## Results and discussion

The optical behavior of the photonic crystal slabs was studied by determining their characteristic photonic band structures (PBSs). PBS of these quasi-3D photonic lattices was computed using the MPB (MIT Photonic Bands) package [28]. The computation of slab band structures requires two steps: First, the slab eigenstates are calculated using preconditioned conjugate-gradient minimization of the Rayleigh quotient in a plane-wave basis [29]. Second, the light cone is obtained and overlapped. As the method requires a unit cell to compute the eigenstates, and the slab is only periodic in two dimensions, a z-supercell approach is required, assuming a periodic sequence of slabs separated by enough background regions. In this case, guided eigenstates are unaffected and only no guided modes are disturbed, but since they fall inside the light cone, their frequencies are inconsiderable [6].

**Figure 3:**
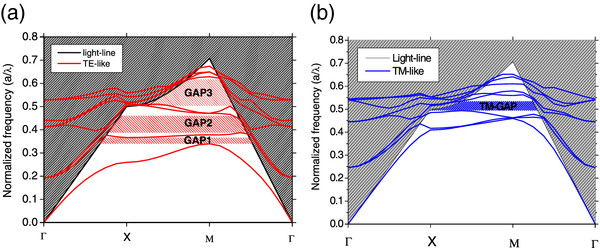
** Photonic band structures corresponding to a square lattice of cylindrical air holes in a Si matrix.** The ratios *r*/*a* and *h*/*a* were set as 0.38 and 0.4, respectively; the parameters correspond to a fluence of 5 × 10^10^/cm and a current density of 300 mA/cm^2^, *ϵ* = 11.56 being the dielectric constant of Si. (**a**) Slabs bands with even symmetry with respect to the z-plane (TE-like). (**b**) Slabs bands with odd symmetry with respect to the z-plane (TM-like).

Figure 3 shows a typical PBS for a square lattice of cylindrical air holes in a Si matrix. In this case, the ratios *r*/*a* and *h*/*a* (where *r* is the radius of the air holes, *a* is the lattice parameter, and *h* is the thickness of the slabs) were set to 0.38 and 0.4, respectively. These parameters correspond to the experimental ones for a fluence of 5 × 10^10^ proton/cm and a current density of 300 mA/cm^2^ (see Tables 1 and 2 for further details). The dielectric constant of Si matrix was set as *ϵ* = 11.56 [30].

**Table 1:** **Experimental ratios (*r*/*a*) for the different combinations of proton fluence and applied current density (*J*)**

**Fluence (proton/cm)**	***J*(mA/cm**^**2**^**)**		
	**3**	**30**	**300**
5e10	0.237	0.295	0.381
8e10	0.213	0.267	0.356
1e11	0.192	0.240	0.308

**Table 2:** **Experimental ratios (*h*/*a*) for the different combinations of proton fluence and applied current density (*J*)**

**Fluence (proton/cm)**	***J*(mA/cm**^**2**^**)**		
	**3**	**30**	**300**
5e10	0.740	0.505	0.397
8e10	0.781	0.565	0.445
1e11	0.813	0.585	0.519

In Figure 3a, several gaps can be observed below the light cone for bands with even symmetry (transverse electric (TE)-like) with respect to reflections through the z-plane (*z* direction being the height slab direction). The first gap opens from the first band to the second band, between 0.342 and 0.366 of the normalized frequency. The second one appears from 0.38 to 0.47 of the normalized frequency, between the second and third band. The last and widest gap opens from the fourth band to the fifth band, between 0.503 and 0.617 of the normalized frequency. In this structure, a gap also appears for bands with odd symmetry with respect to the z-plane (transverse magnetic (TM)-like) below the light cone. This gap appears between the second and third bands in the normalized frequency range from 0.495 to 0.535. This gap shares a range of frequency with the third gap for the TE-like bands. So, this structure has a complete photonic gap between 0.503 and 0.535 of the normalized frequency.

**Figure 4:**
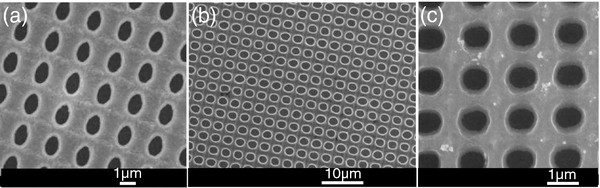
** Scanning electron microscope images of three different samples.** (**a**) Structure with a lattice parameter of 3 μm due to the proton beam line spacing was set to 3 μm. (**b**) Same structure with a lattice parameter of 2 μm. (**c**) The photonic slab presents a period of 1.5 μm.

The frequency ranges where gaps are located depend on the lattice parameter of the structure, since normalized frequency is defined as the ratio between the lattice parameter and the wavelength (normalized frequency = *a*/*λ*). Hence, by controlling the lattice parameter, the frequency range where these structures operate can be tuned. The fabrication process allows control of the lattice parameter by changing the proton beam line spacing when the structure is being irradiated. Figure 4 shows several structures with different lattice parameters. In this case, the irradiated proton lines and line spacing decreased from 3 μm (Figure 4a) to 2 μm (Figure 4b) and 1.5 μm (Figure 4c) to obtain structures with lattice parameters of 3, 2, and 1.5 μm, respectively.

**Figure 5:**
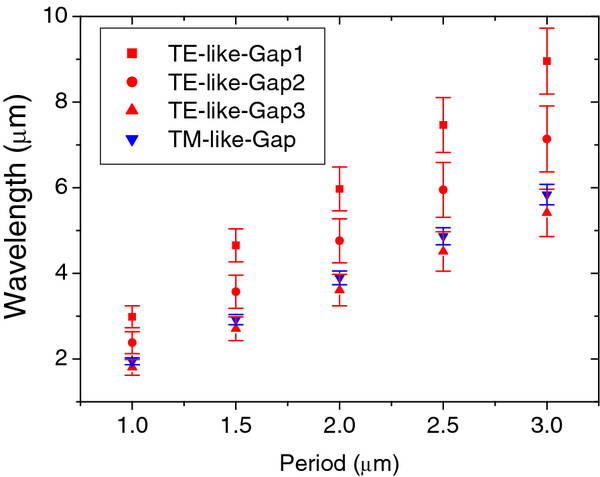
** Theoretical frequency ranges of gaps for square lattices of cylindrical air holes with different lattice parameters.** The ratios *r*/*a* and *h*/*a* were set as 0.38 and 0.4, respectively. The dielectric constant of Si was set at *ϵ* = 11.56.

Figure 5 presents the different frequency ranges of the gaps for several different lattice parameters of the photonic slab. The theoretical results show that the frequency range can be turned over the near-mid infrared range by changing the period of the structure from 1 μm to 3 μm, i.e., by modifying the proton beam line spacing during the irradiation process.

Furthermore, the slabs eigenstates, and consequently their optical properties, strongly depend on the ratios *r*/*a* and *h*/*a.* Once the frequency ranges where the structures operate are determined by fixing the lattice parameter, a fine tuning of these frequency ranges can be accomplished by adjusting the thickness of the slab and radius of the cylindrical air holes. These two parameters can be set by adjusting two main factors in the fabrication process, namely, proton fluence of the beam during the irradiation process and applied current density in the electrochemical anodization process. To study the effect of proton fluence and applied current density on the ratios *r*/*a* and *h*/*a*, lines with some millimeter length were irradiated on Si with different proton fluences. Then, the irradiated areas were electrochemically anodized by applying different current densities.

**Figure 6:**
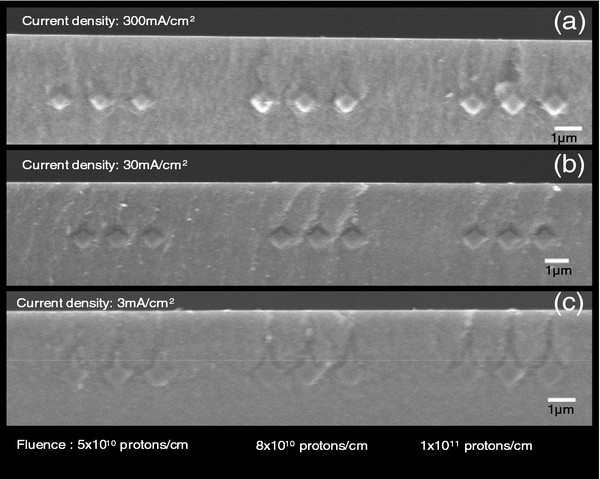
** Cross-sectional SEM image of Si cores with a spacing of 1.5 μm, buried in nanoPSi.** For a 250-keV proton beam, different line fluences were used: 5 × 10^10^/cm, 8 × 10^10^/cm, and 1 × 10^11^/cm in 0.02 Ω cm p-type Si. Subsequent electrochemical etching was carried out using several current densities: (**a**) 300 mA/cm^2^, (**b**) 30 mA/cm^2^, and (**c**) 3 mA/cm^2^.

Figure 6 shows cross-sectional SEM images of buried Si cores, in which 250-keV proton beam was used with three different line fluences: 5 × 10^10^/cm, 8 × 10^10^/cm, and 1 × 10^11^/cm on three different Si wafers. Then, each wafer was anodized by applying three different current densities (3 mA/cm^2^, 30 mA/cm^2^, and 300 mA/cm^2^) with enough time to completely undercut the cores. As noticed in Figure 6, Si core width and core height vary according to the proton fluence and applied current density. For square lattices of air hole slabs, the relationship between the radius of the air holes and the core width is given by *r* = (*a*-core width)/2, whereas the slab thickness is equal to the core height: *h* = core height.

Tables 1 and 2 summarize the experimental values of the ratios *r*/*a* and *h*/*a*, respectively, for the different combinations of proton fluences and anodization current densities. The higher the proton fluence, the larger the core width and core height; thus, the ratio *r*/*a* decreases, whereas *h*/*a* increases for all of the current densities. On the other hand, for a fixed proton fluence, the higher the applied current density, the higher the *r*/*a* and the smaller the *h*/*a*; due to increasing current density, the core width and height become smaller for the same proton fluence.

**Figure 7:**
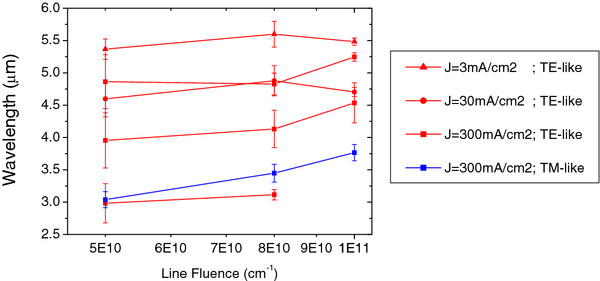
** Computed frequency ranges of the photonic gaps for different line fluences and current densities.** The frequency ranges are for a square lattice of cylindrical air holes in a Si matrix with a fixed period of 1.5 μm.

The effect of varying the proton fluence and applied current density on the optical properties of these structures was studied. Figure 7 shows the frequency ranges of the photonic gaps for the different experimental line fluences and current densities, when the lattice parameter was fixed to 1.5 μm. For a current density of 3 mA/cm^2^, only a TE-like gap appears between the second and third bands. The size of this gap increases from 5% to 8% when the line fluence increases from 5 × 10^10^/cm to 8 × 10^10^/cm. However, an even higher fluence of 1 × 10^11^/cm only provides a 2% gap. The gap size is defined as $\text{Gap}\;\left(\%\right)=\frac{\Delta \omega }{\omega_{c}}\times 100$, where *ω* is the normalized frequency, Δ*ω =* ω_band-n_ − ω_band-n-1_, and *w*_*c*_ is the central frequency of the gap.

For a current density of 30 mA/cm^2^, PBS shows only the same TE-like gap as before, between the second and third band. In this case, the higher the line fluence, the smaller the gap size, being around 12% when the proton fluence is relatively low (5 × 10^10^/cm), decreasing to 10% while increasing fluence to 8 × 10^10^/cm and dropping down to 3% when fluence is further increased to 1 × 10^11^/cm. In these cases, TM-like gaps do not appear yet.

When the current density in the etching process is increased to 300 mA/cm^2^, new gaps open for both symmetries, as Figure 7 shows. For the first TE-like gap, between the first and second bands, its size is reduced as the line fluence is raised, being more than 17% for a low fluence (5 × 10^10^/cm) and dropping down to 3% for the highest fluence (1 × 10^11^/cm). The same behavior appears on the second TE-like gap, between the second and the third bands, showing its maximum size for a low fluence (22%) and its minimum for the highest fluence. Also, in the third TE-like gap, between the fourth and fifth bands, the same behavior is found. However, for the highest fluence (1 × 10^11^/cm), the gap disappears. Furthermore, for this current density, a TM-like gap appears between the third and fourth bands. The size of this gap is almost constant around 8% while increasing the fluence. However, a complete gap for all the symmetries only appears for the lowest line fluence (5 × 10^10^/cm).

As it can be clearly observed in Figure 7, the fabrication process allows a fine tuning of the frequency range at which the gaps open by simply adjusting two main factors, the proton fluence during the irradiation and applied current density in the electrochemical etch. By fixing the lattice parameter to 1.5 μm, TE-like gaps can be turned over a large frequency range over the near-mid infrared, from 2.4 to 6 μm. Moreover, the gap size can be modified too, allowing setting of the frequency range where the gap opens for its proper applications. This process can be extended to the visible and near-IR ranges by decreasing the period and using a suitable proton fluence and applied current density.

Nevertheless, tuning the TM-like gaps is not so straightforward since they only appear for a high current density (typically above 300 mA/cm^2^). However, it can also be modified by changing the proton fluence. Also, a complete gap only appears in some special cases, but due to the flexibility of the method, the experimental parameters can be adjusted for those special cases.

## Conclusion

A highly flexible fabrication process of Si-based photonic slabs for their use as 2D photonic crystals is demonstrated. The process is based on the selective formation of porous silicon by focusing a proton beam on a Si surface, followed by electrochemical etching. The resulting structures are free-standing square lattices of cylindrical air holes embedded in a Si matrix. Their photonic band structures show several gaps below the light cone for the two main directions of symmetry for reflection through the z-plane.

The flexibility of the presented method allows the control of the frequency ranges where the photonic structures can operate by adjusting the proton beam line spacing which tunes the lattice parameter of the structure. Also, a fine tuning of the frequency range can be obtained by adjusting the proton fluence and applied current density, which modify the radius of the air holes and thickness of the slabs. The theoretical results suggest that the fabricated structures represent very promising candidates for the development of Si-based photonic slabs operating in the near-mid infrared ranges.

## Competing interests

The authors declare that they have no competing interests.

## Authors’ contributions

GRS carried out the theoretical studies, analyzed the results, and drafted the manuscript. ZD carried out the fabrication process, participated in the discussion, and helped draft the manuscript. MB assisted in the fabrication process and participated in the discussion. VTC and RJMP helped to analyze the results and draft the manuscript. All authors read and approved the final manuscript.
